# LGP2 Plays a Critical Role in Sensitizing mda-5 to Activation by Double-Stranded RNA

**DOI:** 10.1371/journal.pone.0064202

**Published:** 2013-05-09

**Authors:** Kay S. Childs, Richard E. Randall, Stephen Goodbourn

**Affiliations:** 1 Division of Biomedical Sciences, St. George's, University of London, London, United Kingdom; 2 School of Biology, University of St. Andrews, St. Andrews, United Kingdom; McMaster University, Canada

## Abstract

The DExD/H box RNA helicases retinoic acid-inducible gene-I (RIG-I) and melanoma differentiation associated gene-5 (mda-5) sense viral RNA in the cytoplasm of infected cells and activate signal transduction pathways that trigger the production of type I interferons (IFNs). Laboratory of genetics and physiology 2 (LGP2) is thought to influence IFN production by regulating the activity of RIG-I and mda-5, although its mechanism of action is not known and its function is controversial. Here we show that expression of LGP2 potentiates IFN induction by polyinosinic-polycytidylic acid [poly(I:C)], commonly used as a synthetic mimic of viral dsRNA, and that this is particularly significant at limited levels of the inducer. The observed enhancement is mediated through co-operation with mda-5, which depends upon LGP2 for maximal activation in response to poly(I:C). This co-operation is dependent upon dsRNA binding by LGP2, and the presence of helicase domain IV, both of which are required for LGP2 to interact with mda-5. In contrast, although RIG-I can also be activated by poly(I:C), LGP2 does not have the ability to enhance IFN induction by RIG-I, and instead acts as an inhibitor of RIG-I-dependent poly(I:C) signaling. Thus the level of LGP2 expression is a critical factor in determining the cellular sensitivity to induction by dsRNA, and this may be important for rapid activation of the IFN response at early times post-infection when the levels of inducer are low.

## Introduction

The innate immune system detects invading micro-organisms by sensing the presence of pathogen-specific macromolecules termed pathogen-associated molecular patterns (PAMPs) which display key structural features that identify them as non-self. Mammalian cells express a number of pattern recognition receptors (PRRs) which are responsible for detecting a variety of different PAMPs of bacterial, viral and fungal origin [1]. Their activation stimulates signal transduction pathways that result in innate immune responses including the production of type I interferons (IFN) which play a vital role in controlling infection. Cytoplasmic recognition of RNA viruses is mediated by the retinoic acid-inducible gene (RIG)-I-like receptors, RIG-I, and melanoma differentiation associated gene-5 (mda-5). These PRRs sense distinct, but overlapping RNA structures; RIG-I is activated by short dsRNAs containing a 5′ triphosphate [2]–[4], and although the precise requirements are less clear, mda-5 appears to be activated by longer regions of dsRNA and higher-order RNA structures [5], [6].

RIG-I and mda-5 are characterized by the presence of two N-terminal caspase activation and recruitment domains (CARDs), an RNA helicase domain, and a C-terminal regulatory domain. Recognition of viral RNA occurs through the C-terminal and helicase domains and promotes a conformational change which reveals the CARD domains for downstream signaling [7]. Activation by extended regions of dsRNA is accompanied by the appearance of long filaments formed by co-operative multimerisation of mda-5 or RIG-I along the length of the dsRNA molecule [8]–[10]. In the activated state the CARD domains are exposed and are free to interact with the downstream adapter protein IFN-β promoter stimulator (IPS)-1 (also known as MAVS, Cardif and VISA) which is located on the outer mitochondrial membrane. IPS-1 acts as a scaffold for the assembly of a large multiprotein complex which activates the transcription factors interferon regulatory factor (IRF)-3 and nuclear factor-κB (NF-κB) which are required for transcriptional activation of the IFN-β promoter [11], [12].

Database searches for proteins related to RIG-I identified a factor referred to as laboratory of genetics and physiology 2 (LGP2) [13], [14]. LGP2 shares considerable homology with RIG-I and mda-5 within the RNA helicase and C-terminal domains, but lacks the N-terminal CARD domains that are required for signaling. Consistent with this LGP2 does not have an intrinsic ability to activate the IFN-β promoter in transient overexpression experiments [13].

Interpretation of the relative contributions of RIG-I and mda-5 to IFN induction by specific viruses is complicated by issues such as the presence of virally-encoded inhibitors of PRRs [15], the presence of defective interfering (DI) particles in many virus stocks [16]–[19], and the use of a wide range of cell lines and primary cell types in different studies. Nevertheless, a consensus view is that negative-stranded RNA viruses signal through RIG-I and positive-stranded viruses signal through mda-5, although there are examples of viruses that signal through both [20], [21]. The role of LGP2 in viral infections is less clear. Early experiments showed that overexpression of LGP2 inhibited IFN induction in response to Sendai virus (SeV), Newcastle disease virus (NDV) or polyinosinic-polycytidylic acid [poly(I:C)], a synthetic dsRNA [13], [14], [22] and conversely, that knockdown of LGP2 enhanced activation of an IFN-responsive promoter by NDV. Taken together with the fact that LGP2 is an avid dsRNA binding protein it was proposed that LGP2 inhibits IFN induction by sequestering PAMPs from RIG-I and mda-5 [13], [14] However, studies on LGP2% mice revealed a complex phenotype, which suggested that LGP2 could play positive as well as negative roles in IFN induction. LGP2% mouse embryo fibroblasts (MEFs) produced elevated levels of IFN-β in response to vesicular stomatitis virus (VSV), and the LGP2% mice were more resistant to lethal VSV infection than control mice [23]. In contrast, when these mice were challenged with encephalomyocarditis virus (EMCV), which activates mda-5 rather than RIG-I, they found reduced levels of serum IFN and the mice were less resistant to infection. Thus LGP2 appeared to act as an inhibitor of RIG-I-dependent IFN induction and an activator of mda-5. This study also revealed that the importance of LGP2 may vary between different cell types, since macrophages and bone marrow-derived dendritic cells (BMDCs), but not MEFs, from LGP2% mice showed much lower levels of IFN-β production in response to EMCV than the controls. In more recent work [24], BMDCs from LGP2% mice were found to produce less IFN-β, not just in response to EMCV, but also VSV, SeV, Japanese encephalitis virus (JEV) and Reovirus, which are thought to be activators of RIG-I [20], [25], thus raising the possibility that in some cell types LGP2 may act as a positive regulator of RIG-I as well as mda-5. Interestingly, not all viruses required LGP2 to induce IFN, since IFN induction by influenza A virus was equivalent in the wt and LGP2% cells [24].

To elucidate the role of LGP2 in the cellular response to PAMPs we have studied the ability of LGP2 to influence IFN-β induction by poly(I:C), a molecule generated by the annealing of synthetic single-stranded polyinosinic acid and polycytidylic acid; the annealed material lacks 5′ triphosphate residues and comprises long duplexes of dsRNA joined together by unpaired stretches of polyinosinic acid or polycytidylic acid. These studies are not complicated by undefined viral transcription or replicative structures, the presence of unknown types of DI molecules, or the expression of virally-encoded inhibitors of IFN-β induction. Although previous publications have shown that LGP2 inhibits IFN induction by poly(I:C) when the levels of LGP2 and poly(I:C) are both high [22], we now show that LGP2 is actually a potent stimulator of poly(I:C) signaling when the levels of transfected poly(I:C) are limited, and thus the level of LGP2 is critical in determining cellular sensitivity to induction. We also demonstrate that this function of LGP2 is dependent upon its ability to activate mda-5, and is sensitive to inhibition by the PIV5 V protein. In contrast, although RIG-I can also be activated by poly(I:C), LGP2 does not have the ability to enhance IFN induction by RIG-I, and instead acts as an inhibitor of RIG-I-dependent poly(I:C) signaling when expressed at high levels. Thus, the level of LGP2 expression in a cell may be a crucial factor in shaping the overall IFN response to dsRNA.

## Materials and Methods

### Plasmids

The IFN-β promoter reporter plasmid pIFΔ(−116)lucter [26], the constitutive β-galactosidase reporter plasmid pJatLacZ [27], the expression vector pEFplink2 [28], pEF.mda-5, pEF.RIG-I [29], pEF.Flag.LGP2, pEF.V5.LGP2, pEF.Flag.LGP2(K634E), pEF.Flag.LGP2ΔIV [30], pEF.Flag.RIG-I, pEF.Flag.mda-5Helicase (MH), pEF.mda-5CARD [31] and pEF.PIV5-V [32] have been previously described. pCMVSPORT6.IPS-1 was obtained from the I.M.A.G.E consortium (clone identification no. 5751684) [33]. pEF.Flag.LGP2ΔC which encodes amino acids 1–592 of LGP2, and pEF.Flag.LGP2ΔN which encodes amino acids 145–678 of LGP2 were created using standard methods. To make pEF.Flag.LGP2(K30A) a DNA fragment consisting of the first 183bp of the LGP2 cDNA sequence including a mutation to generate the K30A amino acid change was synthesised by MWG. This was used to replace the corresponding sequence in pEF.Flag.LGP2. LGP2 with helicase motif IV replaced with the equivalent sequence from RIG-I (pEF.Flag.LGP2(IV)R) was made by replacing amino acids 369–380 of LGP2 with amino acids 630–640 of RIG-I using a synthetic DNA fragment (MWG). A pLKO.1-puro plasmid expressing an shRNA against human LGP2 was purchased from Sigma-Aldrich (TRCN0000051267).

For yeast two- and three-hybrid assays, cDNAs were cloned into pGBKT7 or pGADT7 (Clontech) for expression of proteins as GAL4 DNA-binding domain (DBD) or GAL4 activation domain (AD) fusions respectively. pGBKT7.mda-5H and pGADT7.mda-5H, encoding the helicase domain (amino acids 287–1025) of mda-5, pGBKT7.LGP2, pGBKT7.LGP2(IV)R, pHON7, pHON7.PKR(1–207), pHON7.PKR.M2(1–207) and pHON7.PIV5-V have been described elsewhere [30], [31]. The cDNA encoding LGP2 was cloned between the NcoI and EcoRI sites of pGADT7 to generate pGADT7.LGP2. pGADT7.LGP2(K634E) was created by PCR site-directed mutagenesis using standard methods. pGBKT7.LGP2(1–464) was created by cloning a PciI-NcoI fragment of the LGP2 cDNA into the NcoI site of pGBKT7, and pGBKT7.LGP2(145–678) was created by cloning a BspHI-EcoRI fragment of the LGP2 cDNA between the NcoI and EcoRI sites of pGBKT7.

### Cells, transfections and siRNAs

HEK-293 cells (ATCC CRL-1573) were maintained in Dulbecco's modification of Eagle's medium (DMEM) + 10% fetal bovine serum (FBS) and penicillin/streptomycin. Transfections were carried out using linear polyethyleneimine (PEI), MW∼25,000 (Polysciences Inc, Warrington PA, USA) or Lipofectamine (InVitrogen) under standard conditions. Luciferase and β-galactosidase assays were carried out 48h after transfection, and luciferase activity was corrected to β-galactosidase activity. Experiments were repeated at least three times, and average values are presented with error bars indicating the range of values obtained from the replicates. The siRNA against mda-5 is Hs_IFIH1_2 (Qiagen cat no S100445851), the RIG-I siRNA is Hs_DDX58_1 (Qiagen cat no S100361809), and the control siRNA used was against an unrelated protein LRRC37A (Qiagen cat no S100622874). The synthetic double-stranded RNA, polyinosinic-polycytidylic acid [poly(I:C)] was purchased from GE Healthcare Lifesciences (Cat. 27-4732-01), and dissolved in PBS to make a working solution of 1mg/ml which was stored at −20°C. The average length of RNA duplex in the single batch of poly(I:C) used in these experiments was determined by nuclease resistance to range from 200bp to 1000bp with a mean of 500bp. Duplexes are connected by single-stranded regions of RNA of indeterminate length, generating a set of molecules that average over 10kb when compared with dsDNA markers. IFN treatments and inductions with poly(I:C) were carried out as described previously [31].

### Co-immunoprecipitations and immunoblotting

To make cell extracts, 6cm dishes of cells were washed with PBS then lysed in 500 µl lysis buffer (50 mM Tris-HCl pH 7.5, 150 mM NaCl, 1 mM EDTA, 1% NP-40). 100 µl extract was used for co-immunoprecipitation assays using a mouse monoclonal antibody against the Flag tag (Sigma F3165). Complexes were collected on protein A-Sepharose beads (GE Healthcare), washed three times with 1 ml lysis buffer, and eluted in SDS-PAGE loading buffer. Proteins were separated by SDS-PAGE, and immunoblotting was performed using antibodies to the Flag or V5 tags, α-tubulin (Sigma cat. T9026), mda-5 (Alexis Biochemicals AT113) or LGP2 (Abcam, cat. ab67270). Bound primary antibodies were detected using either HRP-conjugated Sheep Anti-mouse Ig or donkey anti-rabbit whole antibody (GE Healthcare).

### Yeast two- and three-hybrid assays

Combinations of GAL4 DBD and GAL4 AD fusion plasmids were introduced into *Saccharomyces cerevisiae* strain PJ69-4α using standard methods. Double transformants were selected on synthetic dropout (SD) medium lacking leucine and tryptophan (SD-L-W), and subsequently streaked onto SD-L-W medium also lacking histidine (SD-L-W-H) and containing 2 mM 3-aminotriazole (3-AT). Growth was monitored for up to 6 days at 30°C. When the pHON7-derived third plasmid was used, triple transformants were selected on SD medium lacking leucine, tryptophan and uracil (SD-L-W-U), and individual colonies were subsequently streaked onto SD-L-W-U medium also lacking histidine and containing 2 mM 3-AT (SD-L-W-U-H).

## Results

### LGP2 enhances IFN induction in response to poly(I:C)

**Figure 1:**
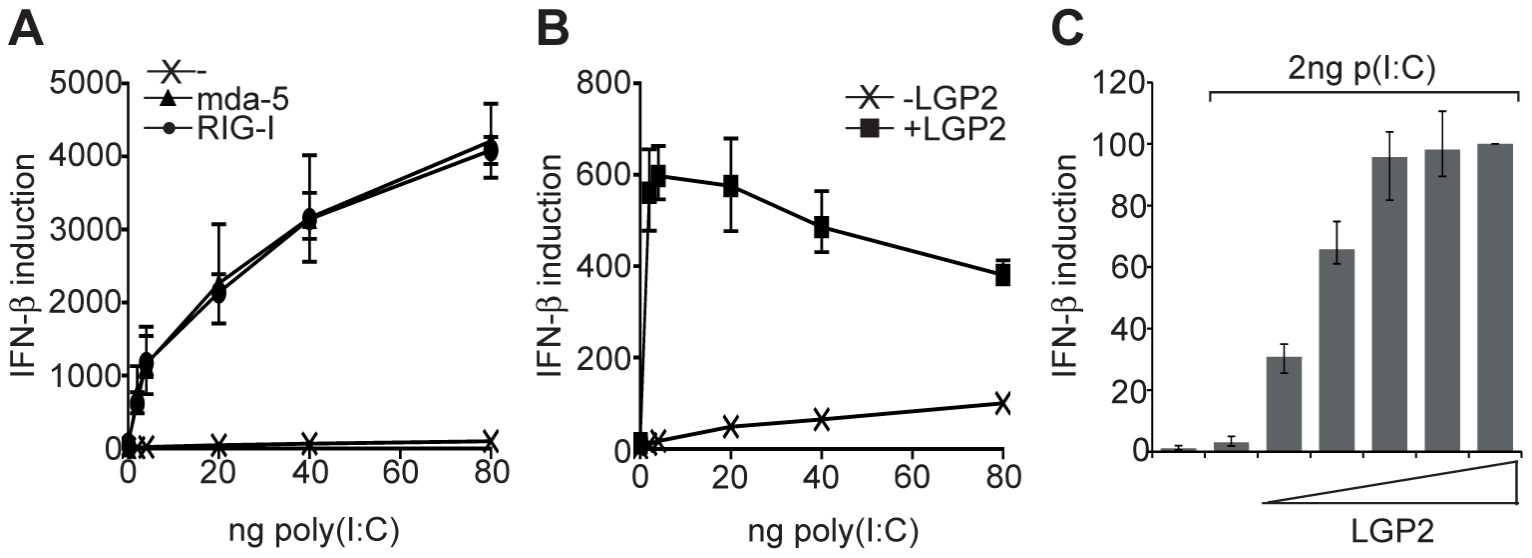
LGP2 enhances IFN induction in response to poly(I:C). (A–C) HEK293 cells were transfected with a reporter plasmid containing the luciferase gene under the control of the IFN-β promoter, a plasmid constitutively expressing β-galactosidase as a transfection control, and (A) 0.4 ng plasmids expressing mda-5 or RIG-I, (B) 100 ng plasmid expressing LGP2, or (C) 0–160 ng plasmid expressing LGP2. Total amounts of DNA were kept constant by supplementing with the empty vector pEFpl2. 24 hours after transfection cells were further transfected with the indicated amounts of poly(I:C) for 16 hours. Cell lysates were analysed for luciferase and β-galactosidase activity, and relative expression levels calculated. The effect of LGP2 on induction by poly(I:C) is statistically significant (p<0.01).

Overexpression of either mda-5 or RIG-I significantly increases IFN induction in response to the synthetic dsRNA, poly(I:C), in HEK293 cells (Fig 1A). Under these conditions the sensitivity to activation of mda-5 or RIG-I by poly(I:C) is indistinguishable. To examine the properties of LGP2, we looked at the effect of LGP2 overexpression on poly(I:C) signaling and found that it could also enhance IFN induction (Fig 1B), but to a lower level than mda-5 and RIG-I (note the different scales in Figs 1A and 1B). Interestingly, this effect was most pronounced at lower levels of transfected poly(I:C). Next, we analysed the effect of increasing amounts of LGP2 on activation of the IFN-β promoter by a fixed amount of poly(I:C). For this experiment we chose to use a low dose of poly(I:C) (2 ng) that barely activated the IFN-β promoter over the uninduced control in the absence of exogenous LGP2 in order to better reveal the effect of LGP2 expression. We found that transfection of cells with a plasmid expressing LGP2 resulted in a considerable dose-dependent increase in the level of IFN induction (Fig 1C). These experiments show that LGP2 is a potent stimulator of poly(I:C) signaling, particularly when the amounts of the inducer are limited.

### LGP2 stimulation of poly(I:C) signaling is dependent upon endogenous mda-5

**Figure 2:**
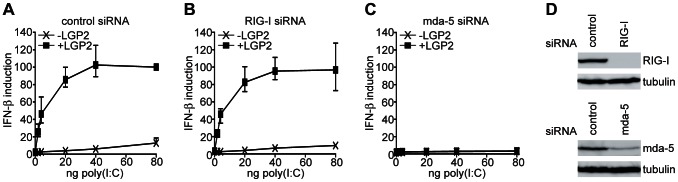
LGP2 stimulation of poly(I:C) signaling is dependent upon endogenous mda-5. HEK293 cells were transfected with the IFN-β reporter plasmid, the β-galactosidase expression plasmid, either the empty vector pEFpl2 or pEF.LGP2, and either a control siRNA (A), an siRNA directed against RIG-I (B), or an siRNA directed against mda-5 (C). 24 hours after transfection cells were further transfected with the indicated amounts of poly(I:C) for 16 hours. Cell lysates were analysed for luciferase and β-galactosidase activity, and relative expression levels calculated. (D) The effectiveness of siRNAs against RIG-I and mda-5 was tested by immunoblotting. HEK293 cells were transfected with a vector expressing Flag-tagged RIG-I and either a control siRNA or the RIG-I siRNA (upper panel). Cells transfected with either the control siRNA or the mda-5 siRNA were exposed to IFN for 16hrs to induce mda-5 expression (lower panel). Cell extracts were subjected to immunoblotting with either anti-Flag (for RIG-I detection), anti-mda-5 or anti-tubulin as a loading control.

Since LGP2 lacks a CARD domain, we hypothesized that the ability of LGP2 to promote poly(I:C) signaling would be dependent upon endogenous mda-5 or RIG-I. To test this we performed the experiment in the presence of either a control siRNA (Fig 2A), a RIG-I siRNA (Fig 2B) or an mda-5 siRNA (Fig 2C). The effectiveness of these siRNAs in knocking down expression of RIG-I and mda-5 in HEK293 cells is shown in Fig 2D. These experiments show that LGP2 is capable of promoting poly(I:C) signaling in the presence of the control or the RIG-I siRNAs, but no induction is observed in the presence of the mda-5 siRNA. Therefore, mda-5 but not RIG-I, is required for the increased IFN induction by poly(I:C) observed upon overexpression of LGP2.

**Figure 3:**
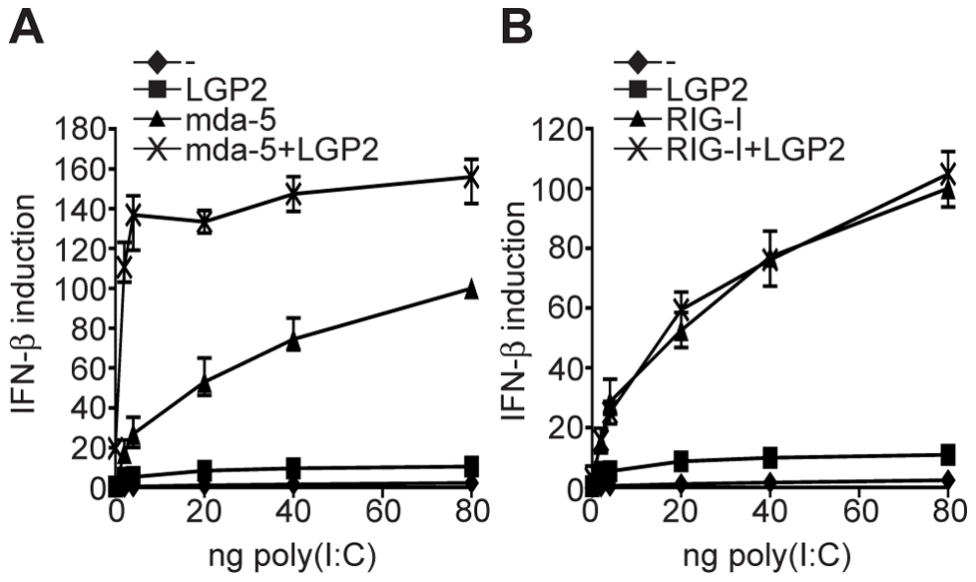
LGP2 co-operates with mda-5 but not RIG-I to promote poly(I:C) signaling. HEK293 cells were transfected with the IFN-β reporter plasmid, the β-galactosidase expression plasmid, and combinations of plasmids expressing mda-5 (0.4 ng), RIG-I (0.4 ng) or LGP2 (2 ng). 24 hours after transfection cells were further transfected with increasing amounts of poly(I:C) for 16 hours. Cell lysates were analysed for luciferase and β-galactosidase activity, and relative expression levels calculated.

We next looked to see if LGP2 expression affects the ability of mda-5 and RIG-I to be activated by poly(I:C). Strikingly, when LGP2 was co-expressed with mda-5, the maximum level of IFN promoter activity was reached at a much lower concentration of poly(I:C) than in the presence of either mda-5 or LGP2 alone (Fig 3A), indicating that LGP2 is able to synergize with mda-5 to render cells considerably more sensitive to induction by poly(I:C). In contrast, LGP2 did not enhance the ability of RIG-I to induce IFN in response to poly(I:C) (Fig 3B). In fact, LGP2 is known to act as an inhibitor of RIG-I signaling, but it has been shown that this requires considerably more LGP2 than activation of mda-5 [30], [34]. Accordingly, we found that higher levels of LGP2 expression inhibited RIG-I-dependent poly(I:C) signaling (Fig S1).

### LGP2 is required for optimal mda-5-dependent IFN induction

**Figure 4:**
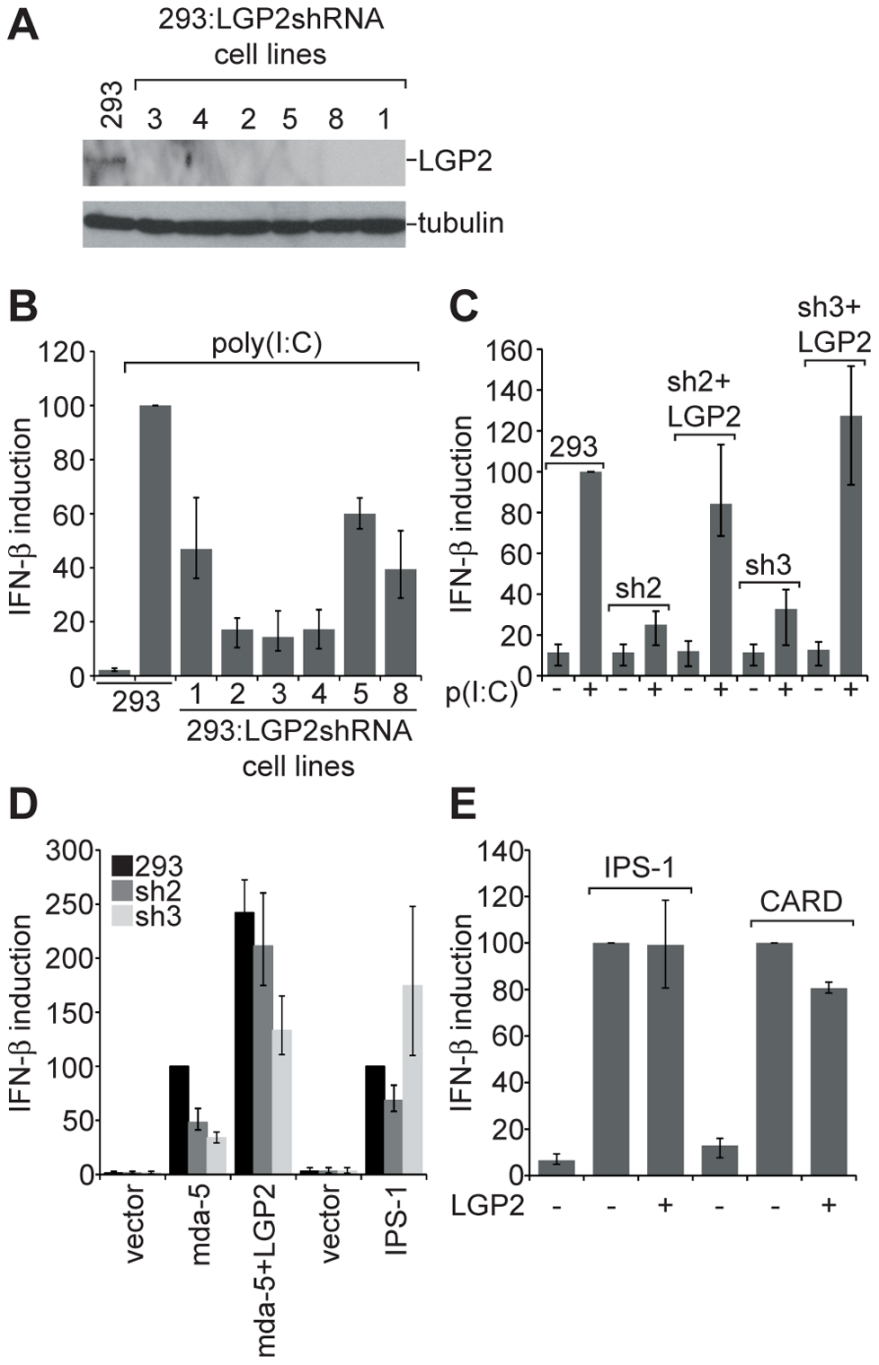
LGP2 knockdown cells induce less IFN in response to poly(I:C) than wild-type cells. (A) Extracts from parental HEK293 cells and stable cell lines expressing an shRNA against LGP2 were subjected to western blotting with antibodies against LGP2 or α-tubulin as a loading control. (B) Parental HEK293 cells and LGP2 knockdown cells were transfected with the IFN-β reporter plasmid and the β-galactosidase expression plasmid. 24 hours after transfection cells were further transfected with poly(I:C) for 16 hours. (C) Parental HEK293 cells and LGP2 knockdown cells were transfected with the IFN-β reporter plasmid, the β-galactosidase expression plasmid, and either the empty vector pEFpl2 or pEF.LGP2. 24 hours after transfection cells were further transfected with poly(I:C) for 16 hours where indicated. (D) Parental HEK293 cells and LGP2 knockdown cells were transfected with the IFN-β reporter plasmid, the β-galactosidase expression plasmid, and plasmids expressing mda-5, LGP2 or IPS-1. (E) HEK293 cells were transfected with the IFN-β reporter plasmid, the β-galactosidase expression plasmid and plasmids expressing IPS-1, the CARD domains of mda-5 or LGP2 as indicated. Cell lysates were analysed for luciferase and β-galactosidase activity, and relative expression levels calculated.

Although it is clear that LGP2 requires mda-5 to promote poly(I:C) signaling, presumably due to the absence of a CARD domain in LGP2, it is not clear whether the efficient activation of mda-5 seen in response to poly(I:C) requires LGP2. We therefore generated stable cell lines expressing an shRNA against LGP2 in order to study the requirement for LGP2 in mda-5-dependent IFN induction. Knockdown of LGP2 in six independent cell lines was verified by western blotting (Fig 4A). Firstly, we examined the ability of these cell lines to induce IFN in response to poly(I:C), and found that in comparison to the parental HEK293 cells the LGP2 knockdown cells responded very poorly (Fig 4B). Two of these cell lines (sh2 and sh3) which showed the least responsiveness to poly(I:C) were chosen for further analysis. To confirm that the defect in IFN induction was in fact due to a lack of LGP2, we overexpressed LGP2 and confirmed that the response to poly(I:C) could be fully restored in both cell lines (Fig 4C). We then looked at IFN induction in these lines in response to mda-5 overexpression, and showed that the level of induction was at least 50% lower in the shRNA-expressing cells than in the control HEK293 cells (Fig 4D). Again this could be restored by overexpression of LGP2. Normal levels of IFN induction were observed in response to overexpression of IPS-1, indicating that these cells do not have a defect in the signaling pathway downstream of mda-5 which could explain the lack of IFN induction. These data show that LGP2 is required for optimal IFN induction through mda-5. Furthermore, overexpression of LGP2 had no effect on IFN induction by IPS-1 or by the isolated CARD domains of mda-5 (Fig 4E), indicating that mda-5 itself, and more specifically the helicase domain of mda-5, is the point of action of LGP2.

### Analysis of the domains of LGP2 required for stimulation of poly(I:C) signaling

**Figure 5:**
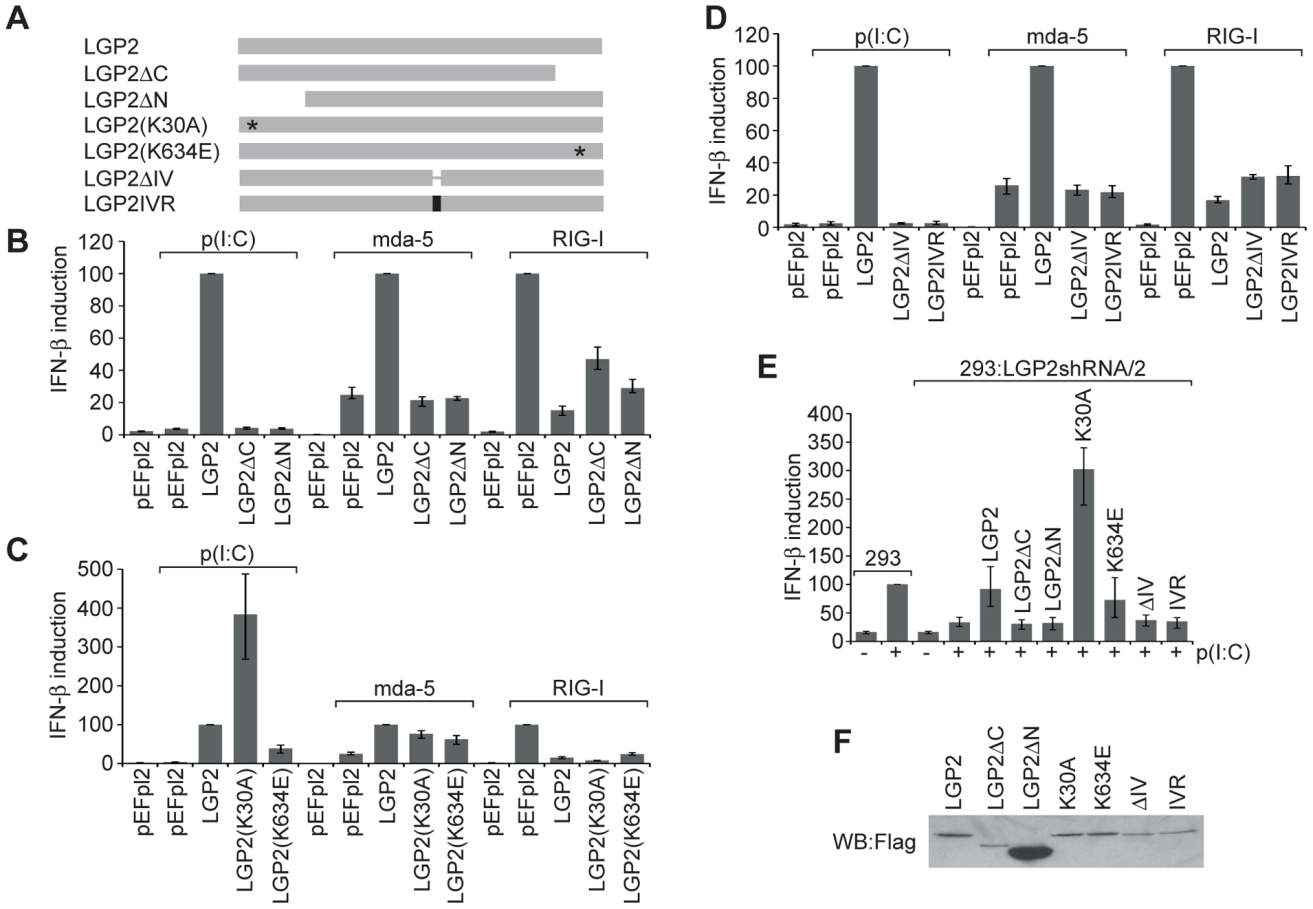
Analysis of the regions of LGP2 required for stimulation of poly(I:C) signaling. (A) Schematic diagram of LGP2 mutants. (B–E) HEK293 cells or LGP2 knockdown cells were transfected with the IFN-β reporter plasmid, the β-galactosidase expression plasmid, and plasmids expressing mda-5, RIG-I, LGP2, or various mutants of LGP2. 24 hours after transfection cells were further transfected with poly(I:C) for 16 hours where indicated. Cell lysates were analysed for luciferase and β-galactosidase activity, and relative expression levels calculated. The average of three independent experiments is shown. (F) HEK293 cells were transfected with plasmids expressing Flag-tagged mutants of LGP2. Lysates of transfected cells were subjected to western blotting using an anti-Flag antibody to confirm expression.

To analyse the domains of LGP2 required to stimulate poly(I:C) signaling, we generated plasmids expressing altered forms of the protein (Fig 5A). Removal of the C-terminal 86 amino acids (LGP2ΔC) or the N-terminal 144 amino acids (LGP2ΔN) inactivated the ability of LGP2 to promote IFN induction in response to poly(I:C), or to stimulate mda-5 (Fig 5B). These altered forms of LGP2 were also unable to rescue the ability of cell lines expressing an LGP2 shRNA to respond to poly(I:C) (Fig 5E). Both truncations retained the ability to inhibit RIG-I exhibited by intact LGP2(Fig 5B).

We also generated two single amino acid substitution forms of LGP2. One of these, LGP2(K30A), lacks ATPase activity but still binds dsRNA [35]; the other, LGP2(K634E), is unable to bind dsRNA [34], [36]. LGP2(K30A) stimulated IFN induction in response to both poly(I:C) and mda-5 overexpression (Fig 5C), and was also able to rescue induction by poly(I:C) in the LGP2 knockdown cells (Fig 5E), indicating that the ATPase activity of LGP2 is dispensable for stimulation of signaling through mda-5 by poly(I:C). By contrast, LGP2(K634E) showed impaired ability to stimulate poly(I:C) or mda-5 signaling (Fig 5C), and only partially rescued the phenotype of the cell lines expressing the LGP2 shRNA (Fig 5E), indicating that the dsRNA binding activity of LGP2 is important for the ability of LGP2 to promote signaling through mda-5. Consistent with previous reports, both LGP2(K30A) and LGP2(K634E) were able to inhibit RIG-I [35], [36].

The helicase domains of mda-5, RIG-I and LGP2 are characterized by the presence of six motifs designated I–VI. We have previously demonstrated that a twelve amino acid region encompassing motif IV, which is completely conserved between mda-5 and LGP2 but which contains 6 amino acid differences in RIG-I, is critical for binding of the paramyxovirus V proteins to mda-5 and LGP2 [30]. To determine whether this region plays a role in the co-operation between LGP2 and mda-5, we generated a plasmid expressing LGP2 with a deletion of amino acids 369–380 which encompasses motif IV (LGP2ΔIV). This completely abolished the ability of LGP2 to stimulate IFN induction in response to both poly(I:C) and mda-5 (Fig 5D). We also introduced a more subtle change by substituting amino acids 369–380 with the equivalent region of RIG-I. This protein (LGP2[IV]R) also failed to stimulate mda-5, indicating that amino acids 369–380 of LGP2 are crucial to the co-operation between mda-5 and LGP2. In addition, neither LGP2ΔIV or LGP2(IV)R were able to rescue the ability of the LGP2 knockdown cells to respond to poly(I:C) (Fig 5E). Interestingly, like the other mutants, deletion of motif IV had no effect on the ability of LGP2 to inhibit IFN induction through RIG-I, demonstrating that the mechanisms that are responsible for mda-5 activation and RIG-I inhibition are distinct and separable.

### LGP2 interacts with mda-5 in a dsRNA-dependent manner

**Figure 6:**
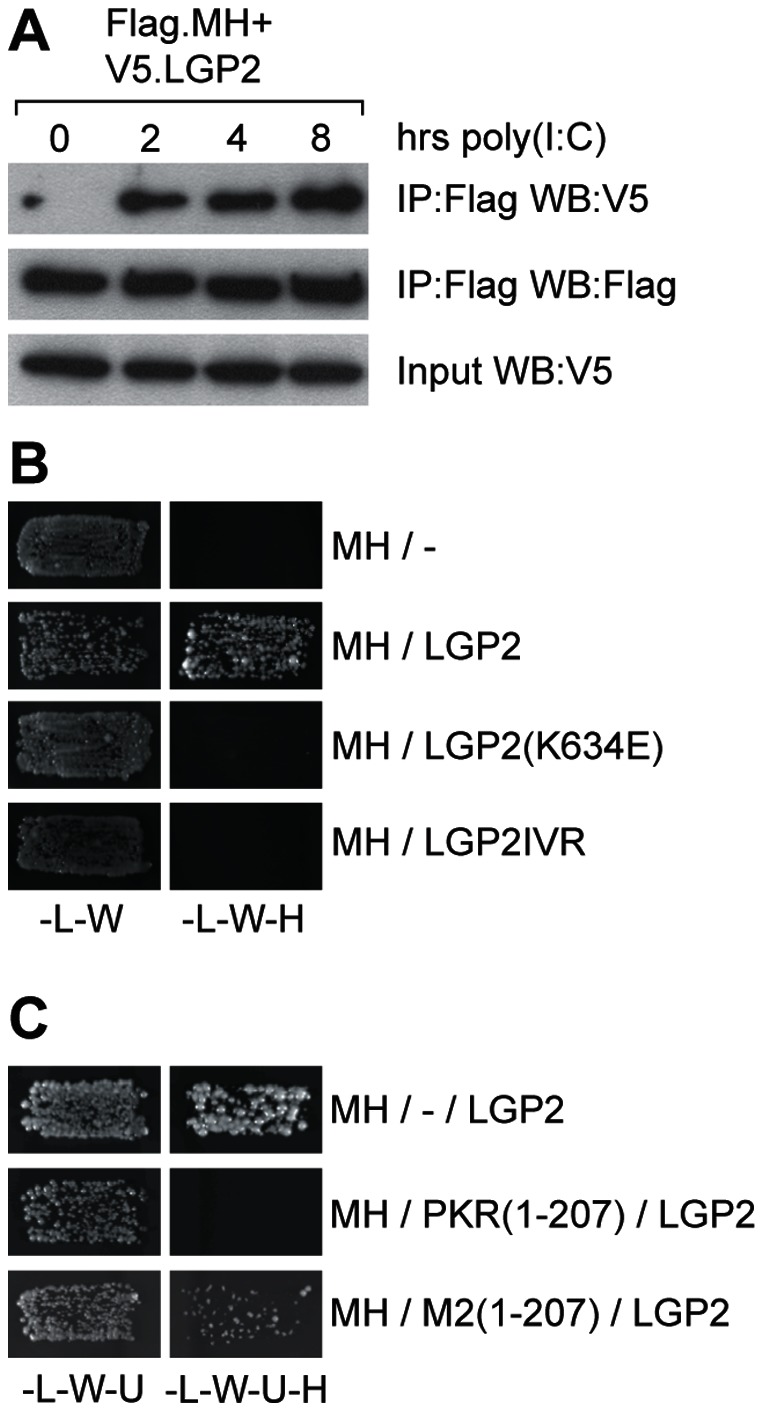
LGP2 interacts with mda-5 in a dsRNA-dependent manner. (A) HEK293 cells were transfected with a plasmid expressing the helicase domain of mda-5 with a Flag tag (Flag.MH) and a plasmid expressing LGP2 with a V5 tag (V5.LGP2). 24 hours after transfection, cells were transfected with poly(I:C) for the indicated times. Cell extracts were subjected to immunoprecipitation with the anti-flag antibody, and proteins present in the precipitate were analysed by western blotting with anti-V5 and anti-flag antibodies. (B) Yeast cells were transformed with a plasmid expressing the helicase domain of mda-5 as a GAL4DBD fusion, and a plasmid expressing LGP2 or the indicated mutants of LGP2 as a GALAD fusion. Positive transformants were selected on SD-L-W, and growth on this media indicates that the yeast have been transformed by both plasmids. They were then streaked onto SD-L-W-H + 2 mM 3-AT and growth on this media demonstrates an interaction between the GAL4DBD fusion protein and the GAL4AD fusion protein. (C) Yeast cells were transformed with a plasmid expressing the helicase domain of mda-5 as a GAL4DBD fusion, a plasmid expressing LGP2 as a GAL4AD fusion and either the empty vector pHON7 (−), pHON7 expressing the dsRNA binding domains of PKR (PKR(1–207)) or pHON7 expressing a mutant form of PKR that is unable to bind dsRNA (M2(1–207)). Positive transformants were selected on SD-L-W-U, and growth on this media indicates that the yeast have been transformed by all three plasmids. They were then streaked onto SD-L-W-U-H + 2 mM 3-AT and growth on this media demonstrates an interaction between the GAL4DBD fusion protein and the GAL4AD fusion protein.

To determine whether the co-operative effect between LGP2 and mda-5 that we observed in the reporter gene assays in response to poly(I:C) is accompanied by a physical association between these two proteins, a co-immunoprecipitation assay was carried out. HEK-293 cells expressing a FLAG-tagged helicase domain of mda-5 and V5-tagged LGP2 were transfected with poly(I:C). No interaction between mda-5 and LGP2 was observed in untreated cells, but upon stimulation with poly(I:C) LGP2 was associated with mda-5 within 2 hours (Fig 6A). This interaction was confirmed in yeast (Fig 6B). We have previously shown that mda-5 can interact with itself in the yeast two-hybrid assay, and that this is dependent on dsRNA present within the yeast strain, because it can be blocked by co-expression of the dsRNA binding domain of PKR (PKR[1–207]; [31]). We therefore repeated this experiment using mda-5 and LGP2, and as we found for mda-5 oligomerisation, the interaction between mda-5 and LGP2 could be blocked by PKR(1–207) but not by a mutant form of PKR which is defective in dsRNA binding activity (M2(1–207)) (Fig 6C). LGP2(K634E) which does not bind dsRNA, did not interact with mda-5, thus confirming the dsRNA-dependence of this interaction. Also, replacement of domain IV of LGP2 with domain IV of RIG-I (LGP2(IV)R) abolished the ability of LGP2 to bind mda-5 (Fig 6B).

### PIV5-V blocks the interaction between mda-5 and LGP2 to inhibit IFN induction

**Figure 7:**
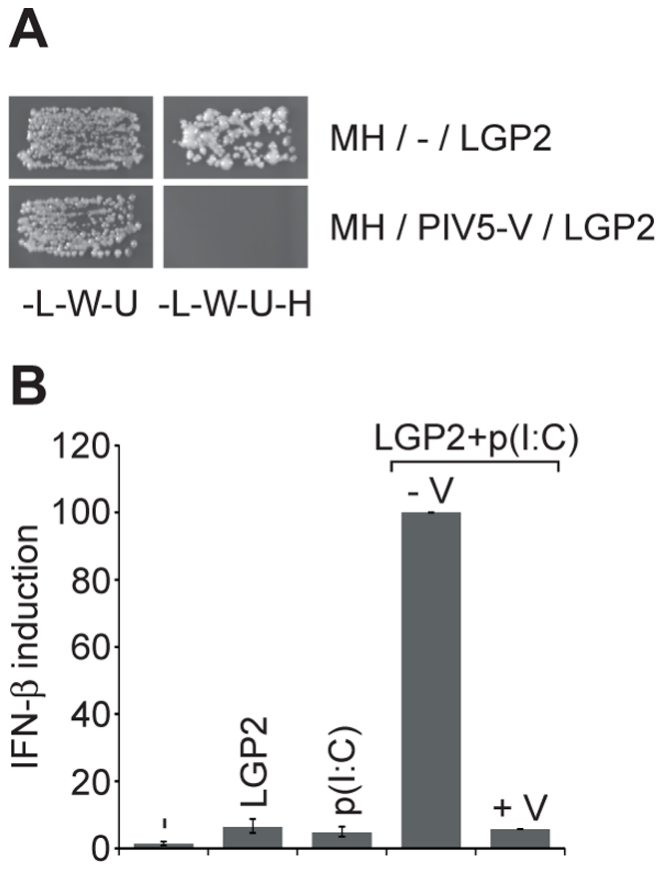
PIV5-V blocks the interaction between mda-5 and LGP2 to inhibit IFN induction. (A) Yeast cells were transformed with a plasmid expressing the helicase domain of mda-5 as a GAL4DBD fusion, a plasmid expressing LGP2 as a GAL4AD fusion and either the empty vector pHON7 (−) or pHON7 expressing the V protein from PIV5 (PIV5-V). Positive transformants were selected on SD-L-W-U and subsequently streaked onto SD-L-W-U-H + 5 mM 3-AT to assay for an interaction between the GAL4DBD fusion protein and the GAL4AD fusion protein. (B) HEK293 cells were transfected with the IFN-β reporter plasmid, the β-galactosidase expression plasmid and either the empty vector pEFpl2 (−), pEF.LGP2 or pEF.PIV5-V as indicated. 24 hours after transfection cells were further transfected with 2 ng poly(I:C) for 16 hours. Cell lysates were analysed for luciferase and β-galactosidase activity, and relative expression levels calculated.

The V protein encoded by members of the *Paramyxovirinae* subfamily of paramyxoviruses can bind to both mda-5 and LGP2 to inhibit IFN induction [30], [37]. V blocks activation of mda-5 by preventing it from oligomerising in the presence of dsRNA, and we have shown that the ability of the mda-5 helicase domain to self-associate in yeast can be blocked by co-expression of the V protein from the paramyxovirus PIV5 (PIV5-V). We therefore used this yeast based assay to determine whether the interaction between mda-5 and LGP2 could also be blocked by PIV5-V, and indeed we found that this was the case (Fig 7A). We also found that PIV5-V was able to block LGP2-dependent IFN induction in the presence of poly(I:C) (Fig 7B).

## Discussion

The data presented here demonstrate that LGP2 acts as a potent stimulator of IFN induction by poly(I:C), and that this effect is particularly significant when very low concentrations of poly(I:C) are used. This indicates that LGP2 is a limiting factor for IFN induction by poly(I:C) in HEK293 cells, a cell line commonly used to study IFN induction. We have shown that the ability of LGP2 to stimulate IFN production is dependent upon endogenous mda-5, and that mda-5 and LGP2 can co-operate to increase the sensitivity of cells to induction by poly(I:C). This, together with the demonstration that mda-5 and LGP2 form a physical association in response to poly(I:C), leads us to propose a model in which a heterodimer or heterooligomer of mda-5 and LGP2 represents a PRR for poly(I:C). This idea is supported by experiments on MEFs from mda-5/LGP2 double knockout mice which fail to make IFN-β in response to EMCV infection. Overexpression of both mda-5 and LGP2 rescued the ability of these cells to respond to EMCV, whereas either one alone was not sufficient [24]. A notable feature of mda-5 activation is the formation of long filaments in which mda-5 dimers co-operatively bind along the length of the dsRNA molecule [9], [10], [38]. In light of the ability of LGP2 to co-operate with mda-5 to induce IFN, a key question that needs to be addressed is whether LGP2 has a role in the formation or the stability of these filaments and whether it also becomes incorporated into the structure.

Although poly(I:C) can activate both mda-5 and RIG-I, we saw no evidence that LGP2 can stimulate poly(I:C) signaling through RIG-I. Our data clearly demonstrate that the co-operative effect observed between LGP2 and mda-5 in the presence of poly(I:C) (Fig 3A), does not occur between LGP2 and RIG-I (Fig 3B). Indeed LGP2 instead acts as an inhibitor of RIG-I, but only when the levels of LGP2 are high. Therefore, if LGP2 does play a negative role in RIG-I signaling *in vivo*, it may only occur in cells in which the levels of LGP2 are in considerable excess over RIG-I. An exception to this occurs in cells infected with paramyxoviruses, where the expressed V protein is able to repress RIG-I in a manner that depends upon binding to LGP2 [30].

LGP2 with a single amino acid substitution which disrupts ATP binding and hydrolysis, LGP2(K30A), retained the ability to stimulate poly(I:C) signaling and mda-5 activity (Fig 5C). However, whereas reconstitution of LGP2% cells with wild-type LGP2 restored their ability to induce IFN in response to EMCV, LGP2(K30A) was ineffective, suggesting that the ATPase activity of LGP2 is required for mda-5-dependent IFN induction by EMCV [24]. Recent work by Bruns et al has shown that LGP2 has a relatively high basal level of ATP hydrolysis, and that this facilitates the recognition of a greater diversity of dsRNA substrates, including molecules that bind relatively weakly to LGP2 in the absence of ATP [39]. Since LGP2 binds poly(I:C) very effectively in a manner that does not depend on ATP, we suggest that there would be no requirement for the ATP hydrolysis activity of LGP2 to stimulate mda-5 or poly(I:C) signaling. Interestingly, LGP2(K30A) was a stronger activator of IFN induction in response to poly(I:C) than wild-type LGP2 (Fig 5C). The reason for this is unclear, but mda-5-catalysed ATP hydrolysis has been linked to the dissociation of mda-5 filaments from dsRNA [9], [38]. Although LGP2 has yet to be linked to the formation of mda-5 filaments, if it is incorporated into the structure, the ATPase activity of LGP2 could also be involved in disassembly. The presence of LGP2(K30A) could therefore result in increased stability of the filaments and consequently lead to increased IFN production.

The dsRNA binding defective form of LGP2(K634E) did not stimulate poly(I:C) signaling as efficiently as wtLGP2 (Fig 5C), suggesting that dsRNA binding is involved in stimulation of poly(I:C) signaling through mda-5. Since the relative affinity of mda-5 for poly(I:C) is low compared to RIG-I and LGP2 (14 and our unpublished data), an attractive model would be one in which LGP2 binds poly(I:C) and helps to recruit mda-5.

We also reveal a critical role for amino acids 369–380 of LGP2 in stimulation of mda-5, since their deletion resulted in a protein which was completely unable to activate IFN induction by poly(I:C) or mda-5. Interestingly, this twelve amino acid region encompassing domain IV is completely conserved between mda-5 and LGP2, but is only 50% identical in RIG-I. Replacement of domain IV of LGP2 with the equivalent sequence from RIG-I resulted in a defective protein, demonstrating that the particular residues common to mda-5 and LGP2 are critical to the ability of LGP2 to stimulate signaling through mda-5. It is interesting to note that this same region is also required for the binding of paramyxovirus V proteins to mda-5 and LGP2 [30], [31], and indeed, we observed that PIV5-V was able to disrupt the interaction between mda-5 and LGP2. It is interesting to speculate that these viruses may have evolved the ability to target this particular region in order to prevent the synergistic effect of their association.

With its important role in regulating activation of mda-5 and thereby determining the sensitivity of cells to induction by mda-5 ligands, and its additional role in providing a brake on RIG-I-dependent IFN induction, the level of LGP2 expression in a cell may actually be a crucial factor in shaping the overall IFN response. Although there is still much work to be done to fully define the role of LGP2 in regulating IFN induction in response to different viral infections, it is clear that this important regulator has a much more complex role than at first envisaged.

## Supporting Information

Figure S1 **At high concentrations, LGP2 inhibits RIG-I-dependent poly(I:C) signaling.** HEK293 cells were transfected with the IFN-β reporter plasmid, the β-galactosidase expression plasmid and plasmids expressing RIG-I (0.4 ng) or LGP2 (100 ng). 24 hours after transfection cells were further transfected with increasing amounts of poly(I:C) for 16 hours. Cell lysates were analysed for luciferase and β-galactosidase activity, and relative expression levels calculated.
(TIF)
