# Supplementary figures and images for: LGP2 Plays a Critical Role in Sensitizing mda-5 to Activation by Double-Stranded RNA
Source: PLoS One. 2013 May 9;8(5):e64202. doi: 10.1371/journal.pone.0064202 (PMC3650065; doi:10.1371/journal.pone.0064202)

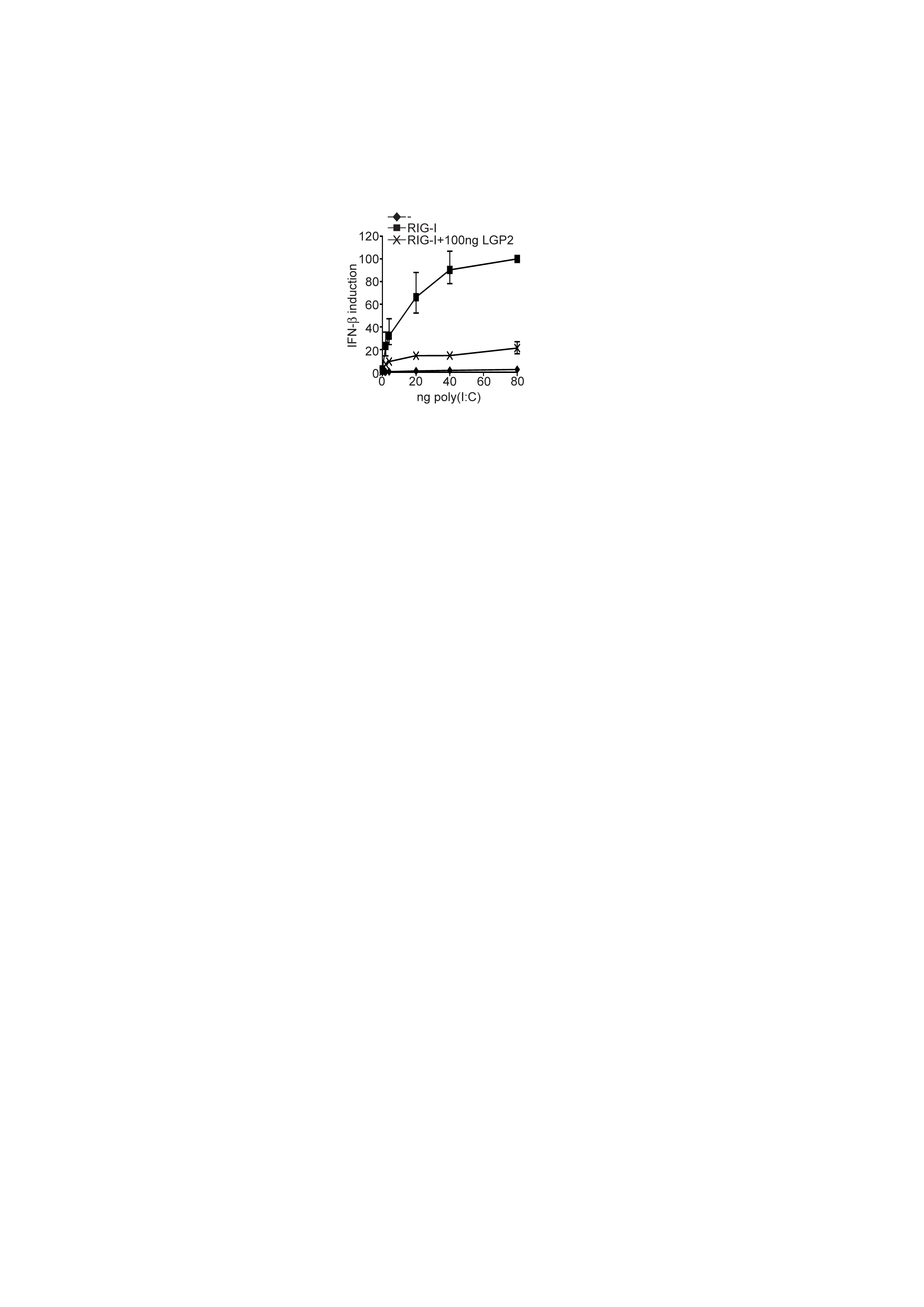

Supplement: Figure S1 — At high concentrations, LGP2 inhibits RIG-I-dependent poly(I:C) signaling. HEK293 cells were transfected with the IFN-β reporter plasmid, the β-galactosidase expression plasmid and plasmids expressing RIG-I (0.4 ng) or LGP2 (100 ng). 24 hours after transfection cells were further transfected with increasing amounts of poly(I:C) for 16 hours. Cell lysates were analysed for luciferase and β-galactosidase activity, and relative expression levels calculated. (TIF) [file pone.0064202.s001.tif]
